# Supplementary figures and images for: Base editing in bovine embryos reveals a species-specific role of SOX2 in regulation of pluripotency
Source: PLoS Genet. 2022 Jul 5;18(7):e1010307. doi: 10.1371/journal.pgen.1010307 (PMC9286228; doi:10.1371/journal.pgen.1010307)

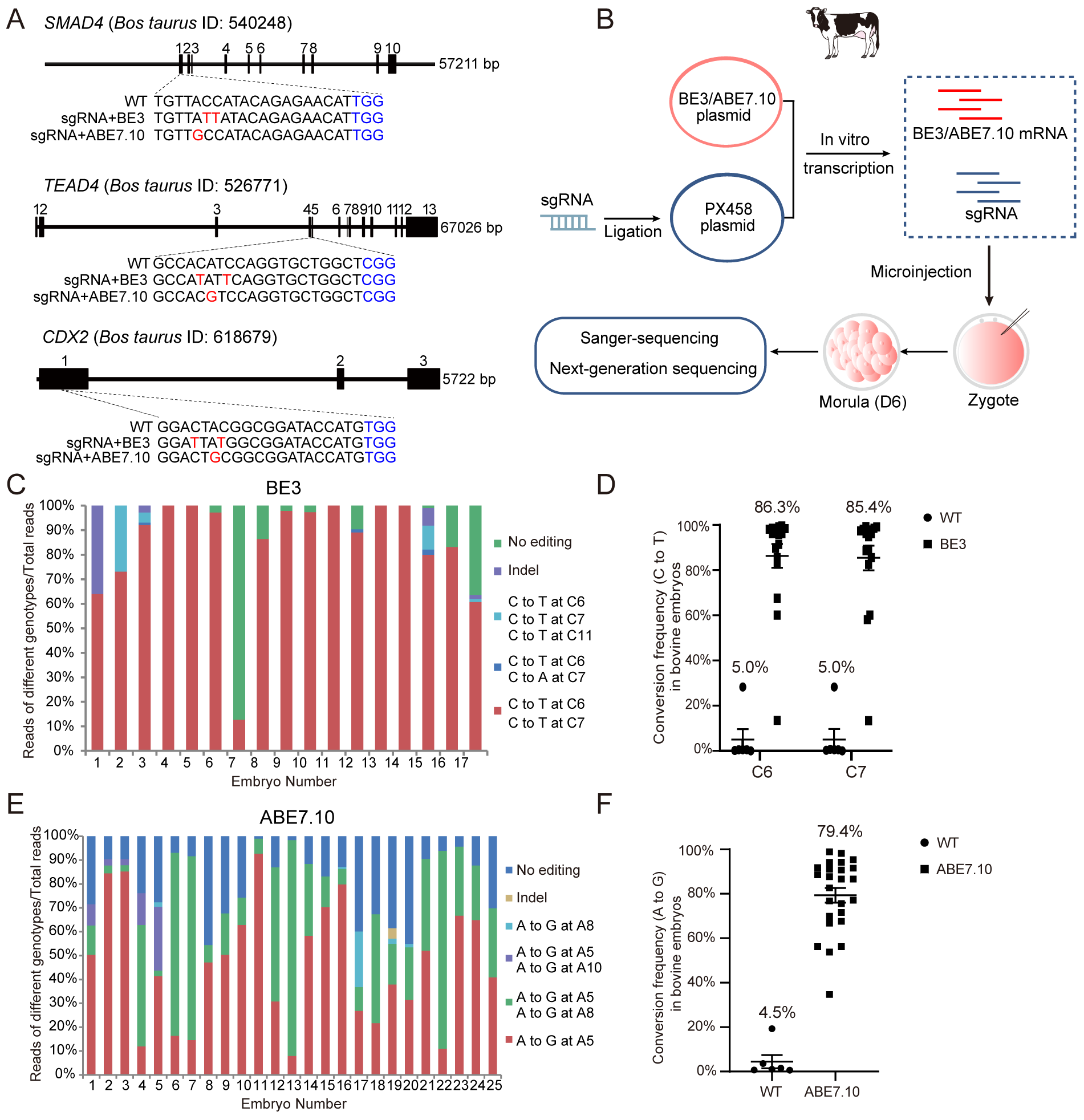

Supplement: S1 Fig — A. Target sites of sgRNA designed for SMAD4, TEAD4, CDX2. Red letters represent the target sites of BE3 or ABE7.10. Blue letters represent protospacer adjacent motif (PAM) sequences. B. Experimental scheme for base editing in bovine early embryos. C and D: Results of targeted deep sequencing for base editing of SMAD4 by BE3 in bovine embryos. 17 embryos were analyzed. E and F: Results of targeted deep sequencing for base editing of SMAD4 by ABE7.10 in bovine embryos. 25 embryos were analyzed. (TIF) [file pgen.1010307.s001.tif]

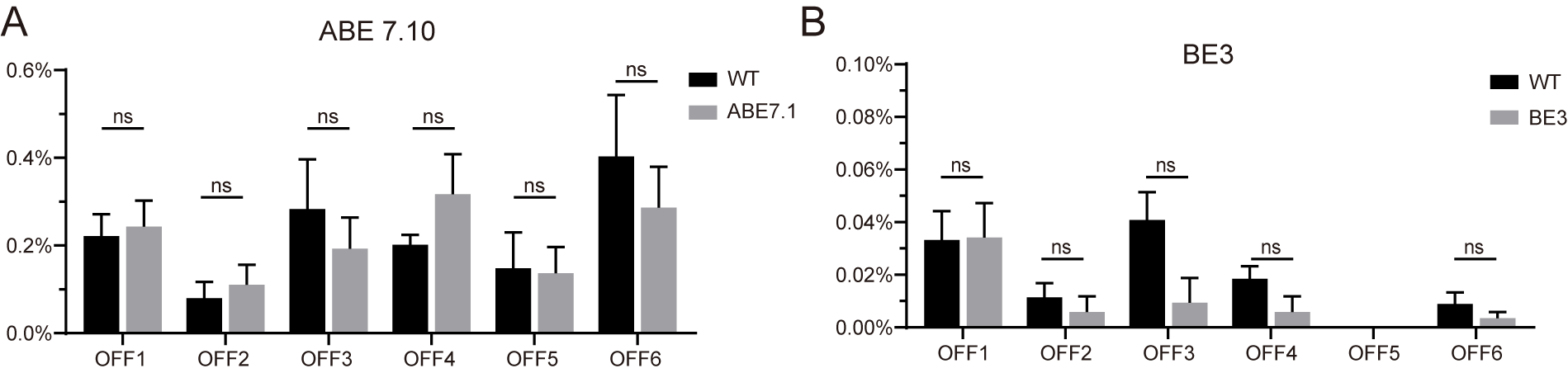

Supplement: S2 Fig — A and B: Targeted deep sequencing analysis of 6 potential off-target sites for ABE7.10 (A) and BE3 (B). (TIF) [file pgen.1010307.s002.tif]

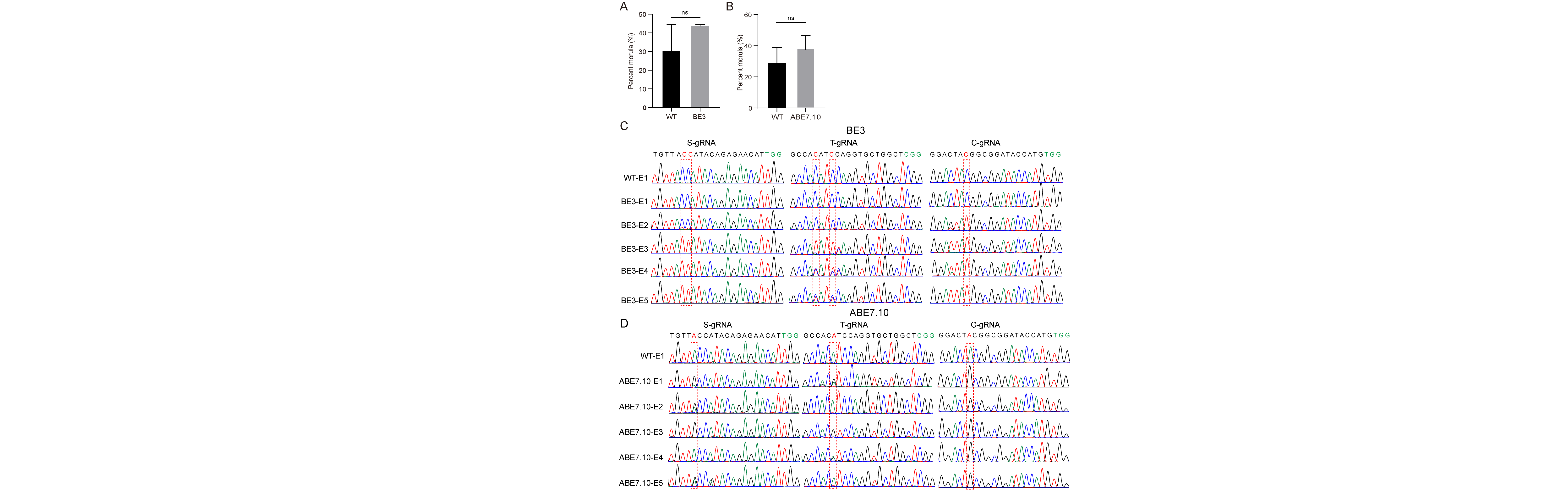

Supplement: S3 Fig — A and B: Embryonic developmental rate to reach morula stage in the WT and ABE7.10 (A) or BE3 (B) group (Two replicates of 13–18 embryos per group). C. Representative Sanger sequencing results of ABE7.10-meidated base editing. D. Representative Sanger sequencing results of BE3-mediated base editing. The red letters and frames represent the editing sites. The green letters represent PAM sequence. S-gRNA: SMAD4 sgRNA; T-gRNA: TEAD4 sgRNA; C-gRNA: CDX2 sgRNA. (TIF) [file pgen.1010307.s003.tif]

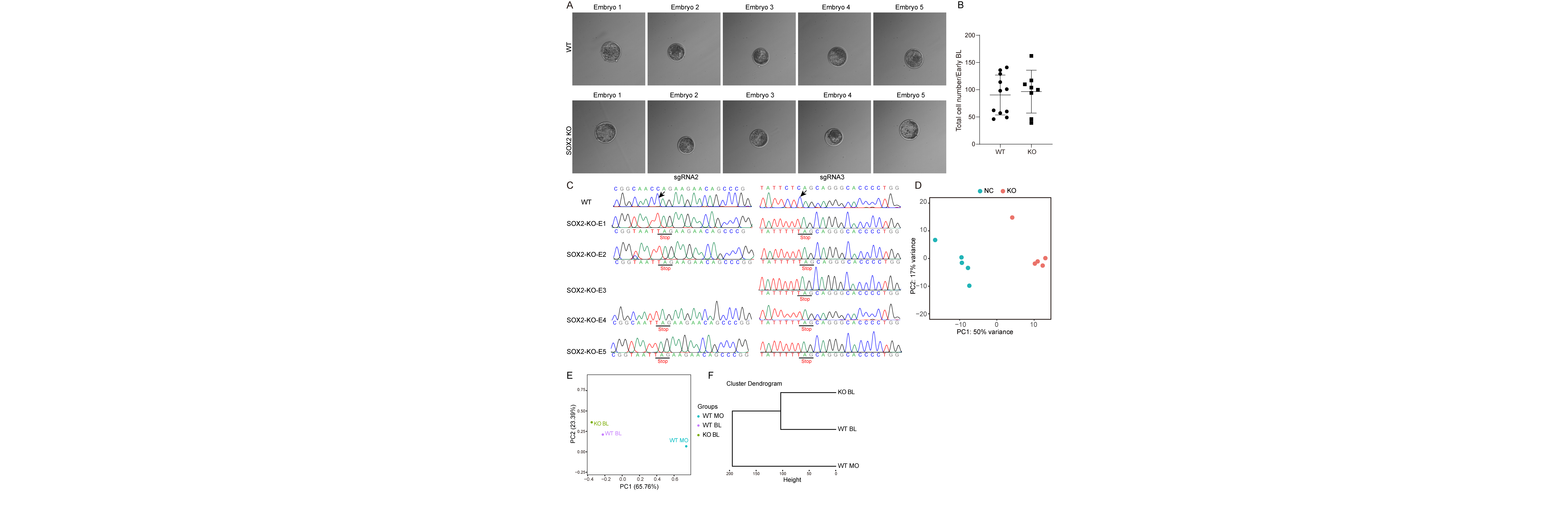

Supplement: S4 Fig — A. Single D7.5 early blastocysts were collected (n = 5 per group) to perform RNA sequencing. B. Early blastocysts collected on D7.5 and used for RNA-seq have similar total cell number. C. Validation of the genotypes of embryos used for RNA-sequencing in A. D. Principal component analysis (PCA) shows high correlation among samples in the same group. E and F. Principal component analysis (E) and hierarchical clustering analysis (F) by comparing the transcriptomes of wildtype (WT BL) and SOX2 KO (KO BL) D7.5 blastocysts with the one of wildtype D6 morula (WT MO, Datasets No.: GSE158679). (TIF) [file pgen.1010307.s004.tif]

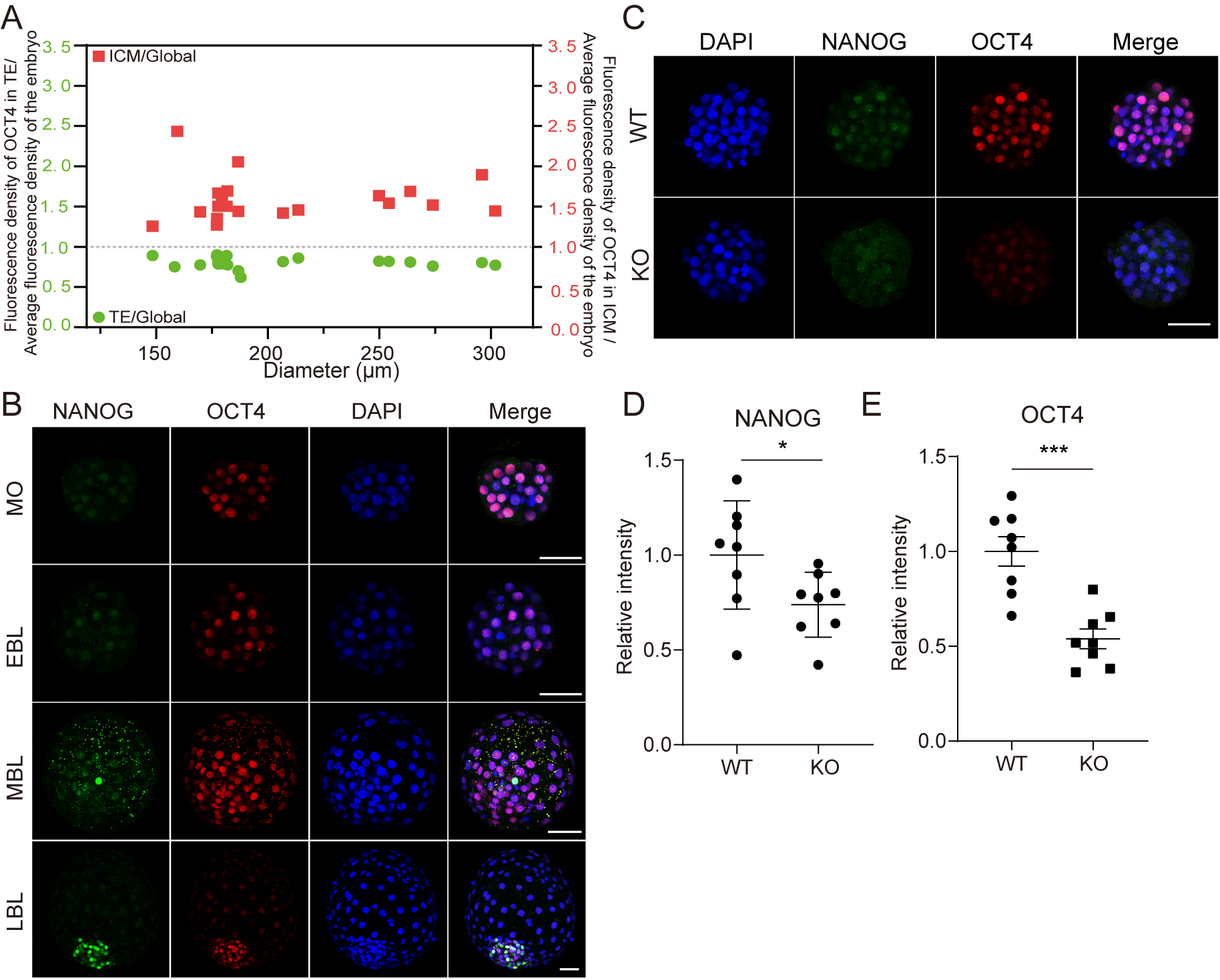

Supplement: S5 Fig — A. Quantification analysis of the changes of OCT4 levels in TE and ICM as blastocyst expansion. TE: SOX2- cells; ICM: SOX2+ cells. B. The dynamics of NANOG and OCT4 expression accompanied by the blastocyst expansion. Green: NANOG; Red: OCT4 (Two replicates of 3–5 blastocysts at different stages). Scale bar = 50 μm C. Immunostaining pictures of NANOG and OCT4 in WT and SOX2 KO (KO) embryos at E6.5. D and E. Relative intensity of NANOG (D) and OCT4 (E) in WT and KO groups. Scale bar = 50 μm. (TIF) [file pgen.1010307.s005.tif]

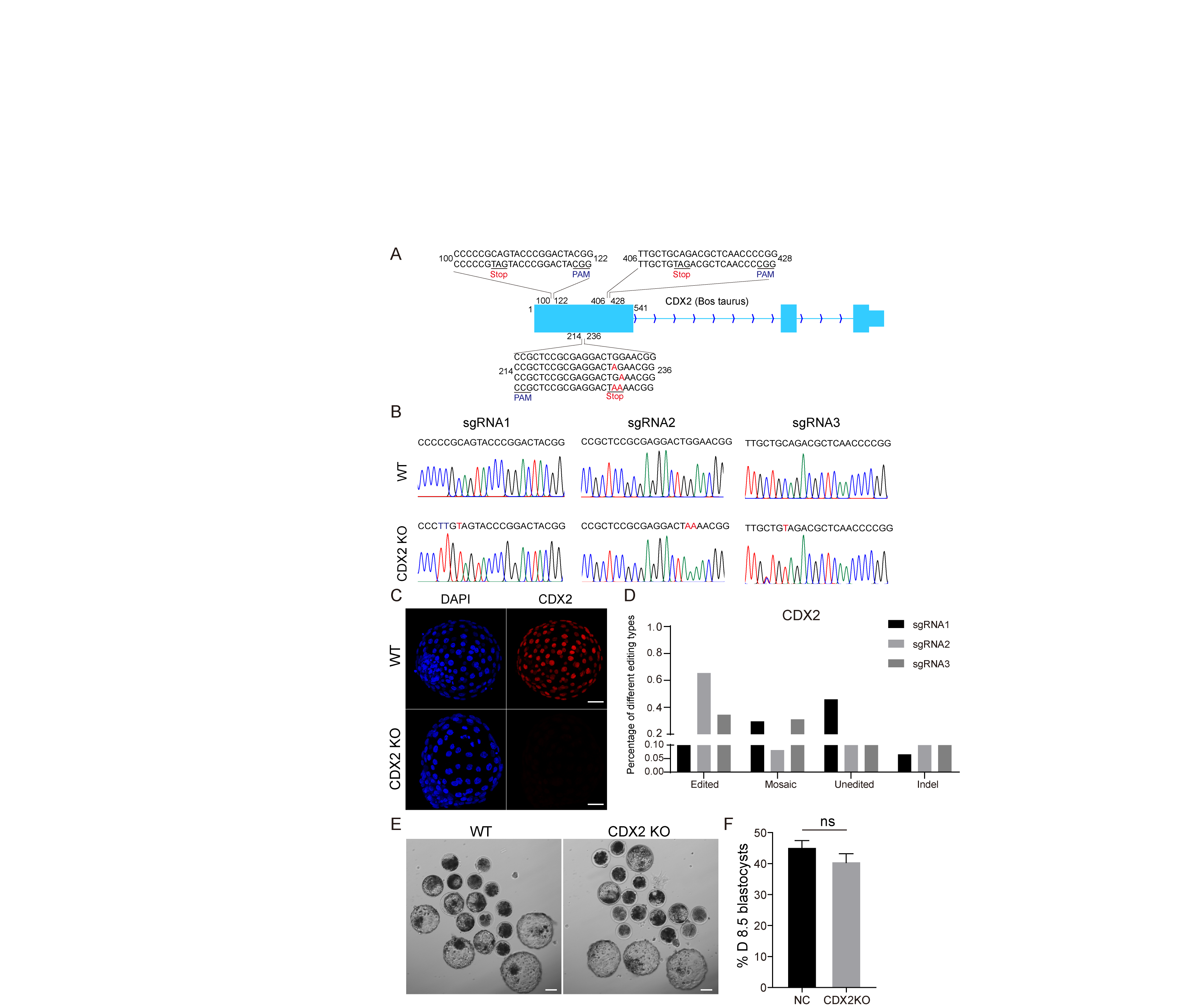

Supplement: S6 Fig — A. sgRNAs designed to target CDX2. B. Representative Sanger sequencing results of CDX2 editing. The red letters represent editing sites. C. Immunostaining detection of CDX2 (Three replicates of 5–8 blastocysts per group). Red: CDX2. Scale bar = 50 μm. D. Editing types analysis using CDX2 sgRNA1, sgRNA2 and sgRNA3 (61 embryos were detected). E and F. CDX2 KO has no effect on the rate of blastocyst formation (Ten replicates of 20–25 embryos per group). Scale bar = 100 μm. (TIF) [file pgen.1010307.s006.tif]
