# Supplementary material for: Base editing in bovine embryos reveals a species-specific role of SOX2 in regulation of pluripotency
Source: PLoS Genet. 2022 Jul 5;18(7):e1010307. doi: 10.1371/journal.pgen.1010307 (PMC9286228; doi:10.1371/journal.pgen.1010307)
Supplement: S1 Table — (PDF) [file pgen.1010307.s007.pdf]

S1_Table. The synthesis of sgRNAs sequence

Gene name	Gene ID	sgRNA	Exon	Sequence（5' – 3'）
SMAD4	540248	S-gRNA	1	FP：CACCTGTTACCATACAGAGAACAT<br>RP：AAACATGTTCTCTGTATGGTAACA
TEAD4	526771	T-gRNA	5	FP：CACCGCCACATCCAGGTGCTGGCT<br>RP：AAACAGCCAGCACCTGGATGTGGC
CDX2	618679	C-gRNA	1	FP：CACCGGACTACGGCGGATACCATG<br>RP：AAACCATGGTATCCGCCGTAGTCC
CDX2	618679	sgRNA1	1	FP：CACCCCCCCGCAGTACCCGGACTA<br>RP：AAACTAGTCCGGGTACTGCGGGGG
CDX2	618679	sgRNA2	1	FP：CACCCCGTTCCAGTCCTCGCGGAG<br>RP：AAACCTCCGCGAGGACTGGAACGG
CDX2	618679	sgRNA3	1	FP：CACCTTGCTGCAGACGCTCAACCC<br>RP：AAACGGGTTAGACGTCTGCAGCAA
OCT4	282316	sgRNA1	1	FP：CACCGCTTCCAAGGGCCTCCCGGT<br>RP：AAACACCGGGAGGCCCTTGGAAGC
OCT4	282316	sgRNA2	1	FP：CACCCCCTCAGCCCGAGGGCGAGG<br>RP：AAACCCTCGCCCTCGGGCTGAGGG
SOX2	784383	sgRNA1	1	FP：CACCCCGCAGCAAACTTCGGGGGG<br>RP：AAACCCCCCCGAAGTTTGCTGCGG
SOX2	784383	sgRNA2	1	FP：CACCCGGCAACCAGAAGAACAGCC<br>RP：AAACGGCTGTTCTTCTGGTTGCCG
SOX2	784383	sgRNA3	1	FP：CACCTATTCTCAGCAGGGCACCCC<br>RP：AAACGGGGTGCCCTGCTGAGAATA
