# Supplementary material for: Base editing in bovine embryos reveals a species-specific role of SOX2 in regulation of pluripotency
Source: PLoS Genet. 2022 Jul 5;18(7):e1010307. doi: 10.1371/journal.pgen.1010307 (PMC9286228; doi:10.1371/journal.pgen.1010307)
Supplement: S2 Table — (PDF) [file pgen.1010307.s008.pdf]

S2_Table. The primers information of sgRNA template for in vitro transcription

sgRNA	Primers' name	Sequence（5' – 3'）
S-gRNA	S-g1-T7-F	TTAATACGACTCACTATAGTGTTACCATACAGAGAACAT
T-gRNA	T-g1-T7-F	TTAATACGACTCACTATAGCCACATCCAGGTGCTGGCT
C-gRNA	C-g1-T7-F	TTAATACGACTCACTATAGGACTACGGCGGATACCATG
CDX2-sgRNA1	C-g2-T7-F	TTAATACGACTCACTATAGCCCCCGCAGTACCCGGACTA
CDX2-sgRNA2	C-g3-T7-F	TTAATACGACTCACTATAGCCGTTCCAGTCCTCGCGGAG
CDX2-sgRNA3	C-g4-T7-F	TTAATACGACTCACTATAGTTGCTGCAGACGCTCAACCC
OCT4-sgRNA1	OCT4-g1-T7-F	TAATACGACTCACTATAGGCTTCCAAGGGCCTCCCGGT
OCT4-sgRNA2	OCT4-g2-T7-F	TAATACGACTCACTATAGGCCCTCAGCCCGAGGGCGAGG
SOX2-sgRNA1	SOX2-g1-T7-F	TAATACGACTCACTATAGGCCGCAGCAAACTTCGGGGGG
SOX2-sgRNA2	SOX2-g2-T7-F	TAATACGACTCACTATAGGCGGCAACCAGAAGAACAGCC
SOX2-sgRNA3	SOX2-g3-T7-F	TAATACGACTCACTATAGGTATTCTCAGCAGGGCACCCC
	sgRNA-R	AAAAGCACCGACTCGGTGCC
