# Supplementary material for: Base editing in bovine embryos reveals a species-specific role of SOX2 in regulation of pluripotency
Source: PLoS Genet. 2022 Jul 5;18(7):e1010307. doi: 10.1371/journal.pgen.1010307 (PMC9286228; doi:10.1371/journal.pgen.1010307)
Supplement: S3 Table — (PDF) [file pgen.1010307.s009.pdf]

**S3_Table. Nested PCR primer sequences for preparing Sanger sequencing samples**

| Gene | Gene ID | Target sgRNAs | Primers' name | Sequence（5' – 3'） |
|---|---|---|---|---|
| *SMAD4* | 540248 | S-gRNA | S-g1-1 | FP：TCTAACAATTTTCCTTGCAAC<br>RP：CCTGTATTGATAATATCTGTGCT |
| | | | S-g1-2 | FP：TCCGAAAGATCAAAATTGCT<br>RP：ACAGTATCTAAAGAGACGGAG |
| *TEAD4* | 526771 | T-gRNA | T-g1-1 | FP：CTGAGAGGGTGCTGTGTCTC<br>RP：AACGTCCAGTCCCAGAGAGT |
| | | | T-g1-2 | FP：GGACCGCACTTCTGTTTAGC<br>RP：TGGGGCTTACAGGGTTACAG |
| *CDX2* | 618679 | C-gRNA<br>CDX2-sgRNA1<br>CDX2-sgRNA2<br>CDX2-sgRNA3 | C-g-1 | FP：ATGGTGAGGTTCGCCGTC<br>RP：GCTTTACACTGAACGCGGCT |
| | | | C-g-2 | FP：TACGTGAGCTACCTCCTGGAC<br>RP：CCCCGCTATCCCCTACTCA |
| *OCT4* | 282316 | OCT4-sgRNA1<br>OCT4-sgRNA2 | OCT4-g-1 | FP：GCCAGAGGTCAAGGCTAGTG<br>RP：CTCACCTGCGGTTCTCTCTT |
| | | | OCT4-g-2 | FP：CGGGACACCTCGCTTCTGAC<br>RP：CTTCGCCTGCTCCCTTCCTG |
| *SOX2* | 784383 | SOX2-sgRNA1<br>SOX2-sgRNA2<br>SOX2-sgRNA3 | SOX2-g-1 | FP：CCCCCAAATTATTCTTCGCCTG<br>RP：CTTTGAAAATGTCTCCCCCG |
| | | | SOX2-g-2 | FP：GCCCGCATGTACAACATGATGG<br>RP：GGGCAGTGTGCCGTTAATGG |
