# Supplementary material for: Base editing in bovine embryos reveals a species-specific role of SOX2 in regulation of pluripotency
Source: PLoS Genet. 2022 Jul 5;18(7):e1010307. doi: 10.1371/journal.pgen.1010307 (PMC9286228; doi:10.1371/journal.pgen.1010307)
Supplement: S4 Table — (PDF) [file pgen.1010307.s010.pdf]

S4_Table. The predicted potential 6 off-target sites for *SMAD4*

Name	Chromosome	Sequence information
OFF 1	2	TGTTACCATAGCAGAGGACAT
OFF 2	23	TTTTACCATAAAGAGAACTT
OFF 3	11	TCTTACCATTCAGAGAAGCAT
OFF 4	7	TGTTTGCATACAGAGAAGAT
OFF 5	9	TGATCCATACAGAGAAGAT
OFF 6	1	TGTTACCAGCCAGAGAACACT
