# Supplementary material for: Base editing in bovine embryos reveals a species-specific role of SOX2 in regulation of pluripotency
Source: PLoS Genet. 2022 Jul 5;18(7):e1010307. doi: 10.1371/journal.pgen.1010307 (PMC9286228; doi:10.1371/journal.pgen.1010307)
Supplement: S5 Table — (PDF) [file pgen.1010307.s011.pdf]

S5_Table. PCR primer sequences for preparing targeted next generation sequencing samples

Amplified sites	Chromosome	Primers' name	Sequence（5' – 3'）
S-gRNA-ON	24	S-g1-ON-F1	ATCACGGGAGAGAGTGAAACATTCGCC
		S-g1-ON-F2	TTAGGCGGAGAGAGTGAAACATTCGCC
		S-g1-ON-F3	ACAGTGGGAGAGAGTGAAACATTCGCC
		S-g1-ON-F4	CAGATCGGAGAGAGTGAAACATTCGCC
		S-g1-ON-F5	TAGCTTGGAGAGAGTGAAACATTCGCC
		S-g1-ON-F6	GGCTACGGAGAGAGTGAAACATTCGCC
		S-g1-ON-R1	CGATGTAAAGAGACGGAGCAAGCATAAA
		S-g1-ON-R2	TGACCAAAAGAGACGGAGCAAGCATAAA
		S-g1-ON-R3	GCCAATAAAGAGACGGAGCAAGCATAAA
		S-g1-ON-R4	ACTTGAAAAGAGACGGAGCAAGCATAAA
		S-g1-ON-R5	GATCAGAAAGAGACGGAGCAAGCATAAA
S-gRNA-OFF1	2	S-g1-OFF1-F1	ATCACGTCTTTGTGCAGAATGGGGGT
		S-g1-OFF1-F2	TTAGGCTCTTTGTGCAGAATGGGGGT
		S-g1-OFF1-F3	ACAGTGTCTTTGTGCAGAATGGGGGT
		S-g1-OFF1-F4	CAGATCTCTTTGTGCAGAATGGGGGT
		S-g1-OFF1-F5	TAGCTTTCTTTGTGCAGAATGGGGGT
		S-g1-OFF1-F6	GGCTACTCTTTGTGCAGAATGGGGGT
		S-g1-OFF1-R1	CGATGTGATGATTCCCGAGTGGGAGC
		S-g1-OFF1-R2	TGACCAGATGATTCCCGAGTGGGAGC
		S-g1-OFF1-R3	GCCAATGATGATTCCCGAGTGGGAGC
		S-g1-OFF1-R4	ACTTGAGATGATTCCCGAGTGGGAGC
		S-g1-OFF1-R5	GATCAGGATGATTCCCGAGTGGGAGC
S-gRNA-OFF2	23	S-g1-OFF2-F1	ATCACGAATTCACATCATGCTTTTGTTTCAT
		S-g1-OFF2-F2	TTAGGCAATTCACATCATGCTTTTGTTTCAT
		S-g1-OFF2-F3	ACAGTGAATTCACATCATGCTTTTGTTTCAT
		S-g1-OFF2-F4	CAGATCAATTCACATCATGCTTTTGTTTCAT
		S-g1-OFF2-F5	TAGCTTAATTCACATCATGCTTTTGTTTCAT
		S-g1-OFF2-F6	GGCTACAATTCACATCATGCTTTTGTTTCAT
		S-g1-OFF2-R1	CGATGTGCACATGCAGAGATATTTTCTAAGT

**Amplified sites**	**Chromosome**	**Primers' name**	**Sequence（5' – 3'）**
		S-g1-OFF2-R2	TGACCAGCACATGCAGAGATATTTTCTAAGT
		S-g1-OFF2-R3	GCCAATGCACATGCAGAGATATTTTCTAAGT
		S-g1-OFF2-R4	ACTTGAGCACATGCAGAGATATTTTCTAAGT
		S-g1-OFF2-R5	GATCAGGCACATGCAGAGATATTTTCTAAGT
S-gRNA-OFF3	11	S-g1-OFF3-F1	ATCACGGGCGCCTTGATTAATACCCC
		S-g1-OFF3-F2	TTAGGCGGCGCCTTGATTAATACCCC
		S-g1-OFF3-F3	ACAGTGGGCGCCTTGATTAATACCCC
		S-g1-OFF3-F4	CAGATCGGCGCCTTGATTAATACCCC
		S-g1-OFF3-F5	TAGCTTGGCGCCTTGATTAATACCCC
		S-g1-OFF3-F6	GGCTACGGCGCCTTGATTAATACCCC
		S-g1-OFF3-R1	CGATGTGCAACTACCGTGAGAGTTCA
		S-g1-OFF3-R2	TGACCAGCAACTACCGTGAGAGTTCA
		S-g1-OFF3-R3	GCCAATGCAACTACCGTGAGAGTTCA
		S-g1-OFF3-R4	ACTTGAGCAACTACCGTGAGAGTTCA
		S-g1-OFF3-R5	GATCAGGCAACTACCGTGAGAGTTCA
S-gRNA-OFF4	7	S-g1-OFF4-F1	ATCACGACCAGACGAGGTACTGTAACTTT
		S-g1-OFF4-F2	TTAGGCACCAGACGAGGTACTGTAACTTT
		S-g1-OFF4-F3	ACAGTGACCAGACGAGGTACTGTAACTTT
		S-g1-OFF4-F4	CAGATCACCAGACGAGGTACTGTAACTTT
		S-g1-OFF4-F5	TAGCTTACCAGACGAGGTACTGTAACTTT
		S-g1-OFF4-F6	GGCTACACCAGACGAGGTACTGTAACTTT
		S-g1-OFF4-R1	CGATGTCCGGAGGAGCAGACTAGTGAT
		S-g1-OFF4-R2	TGACCACCGGAGGAGCAGACTAGTGAT
		S-g1-OFF4-R3	GCCAATCCGGAGGAGCAGACTAGTGAT
		S-g1-OFF4-R4	ACTTGACCGGAGGAGCAGACTAGTGAT
		S-g1-OFF4-R5	GATCAGCCGGAGGAGCAGACTAGTGAT
S-gRNA-OFF5	9	S-g1-OFF5-F1	ATCACGTGAGGAGAATGCCAACTTGTTC
		S-g1-OFF5-F2	TTAGGCTGAGGAGAATGCCAACTTGTTC
		S-g1-OFF5-F3	ACAGTGTGAGGAGAATGCCAACTTGTTC
		S-g1-OFF5-F4	CAGATCTGAGGAGAATGCCAACTTGTTC
		S-g1-OFF5-F5	TAGCTTTGAGGAGAATGCCAACTTGTTC

Amplified sites	Chromosome	Primers' name	Sequence（5' – 3'）
		S-g1-OFF5-F6	GGCTACTGAGGAGAATGCCAACTTGTTC
		S-g1-OFF5-R1	CGATGTCTGTATTTCAAAATGTAGGACCGT
		S-g1-OFF5-R2	TGACCACTGTATTTCAAAATGTAGGACCGT
		S-g1-OFF5-R3	GCCAATCTGTATTTCAAAATGTAGGACCGT
		S-g1-OFF5-R4	ACTTGACTGTATTTCAAAATGTAGGACCGT
		S-g1-OFF5-R5	GATCAGCTGTATTTCAAAATGTAGGACCGT
S-gRNA-OFF6	1	S-g1-OFF6-F1	ATCACGCACGGTCCAGTAGGTTCCCA
		S-g1-OFF6-F2	TTAGGCCACGGTCCAGTAGGTTCCCA
		S-g1-OFF6-F3	ACAGTGCACGGTCCAGTAGGTTCCCA
		S-g1-OFF6-F4	CAGATCCACGGTCCAGTAGGTTCCCA
		S-g1-OFF6-F5	TAGCTTCACGGTCCAGTAGGTTCCCA
		S-g1-OFF6-F6	GGCTACCACGGTCCAGTAGGTTCCCA
		S-g1-OFF6-R1	CGATGTCCTTCCGATTCCACGCAGT
		S-g1-OFF6-R2	TGACCACCTTCCGATTCCACGCAGT
		S-g1-OFF6-R3	GCCAATCCTTCCGATTCCACGCAGT
		S-g1-OFF6-R4	ACTTGACCTTCCGATTCCACGCAGT
		S-g1-OFF6-R5	GATCAGCCTTCCGATTCCACGCAGT
