# Supplementary material for: Base editing in bovine embryos reveals a species-specific role of SOX2 in regulation of pluripotency
Source: PLoS Genet. 2022 Jul 5;18(7):e1010307. doi: 10.1371/journal.pgen.1010307 (PMC9286228; doi:10.1371/journal.pgen.1010307)
Supplement: S6 Table — (PDF) [file pgen.1010307.s012.pdf]

S6_Table. Antibody information

Name	Host	Company	Catalog Number	Application
SOX2	Rat	eBioscience™	14-9811-82	IF (1:200)
CDX2	Mouse	BioGenex	CDX2-88	IF (1:20)
GATA3	Rabbit	Abcam	ab199428	IF (1:200)
OCT4	Rabbit	Abcam	Ab181557	IF (1:200)
NANOG	Mouse	eBioscience™	14-5768-82	IF (1:100)
SOX17	Goat	Bio-techne	AF1924	IF (1:200)
