# Supplementary material for: Base editing in bovine embryos reveals a species-specific role of SOX2 in regulation of pluripotency
Source: PLoS Genet. 2022 Jul 5;18(7):e1010307. doi: 10.1371/journal.pgen.1010307 (PMC9286228; doi:10.1371/journal.pgen.1010307)
Supplement: Attachment — Submitted filename: Response to Reviewers.docx [file pgen.1010307.s013.docx]

**Dear Dr. Marisa S Bartolomei and Dr. Gregory Barsh,**

**We are very grateful to all reviewers and editors for their valuable comments and suggestions. We believe that the comments have been highly constructive and very helpful to improve the quality of the revised manuscript. Thus, we have carefully revised the manuscript by taking into account all the comments from the reviewers. The specific changes made in the revised manuscript are summarized in our point-by-point responses as shown below.**

Editors’ Comments to the Author:

The manuscript was fully evaluated at the editorial level and by independent peer reviewers. The reviewers are quite enthusiastic about this important problem, but two of the reviewers raised some substantial concerns about the current manuscript. Importantly, they would like to see the RNA-seq analysis improved as well as significantly improved immunofluorescence to provide more convincing data on the downregulation of Oct4. Based on the reviews, we will not be able to accept this version of the manuscript, but we would be willing to review a much-revised version. We cannot, of course, promise publication at that time.

**Response: Thank you very much. We have addressed the concern on RNA-seq analysis and immunofluorescence specifically as below.**

Reviewers’ Comments to the Author:

Reviewer 1: This manuscript from K Zhang’s laboratory represents an excellent contribution to the field of early mammalian development and is highly commendable in not only rigorously adapting base editing to gene knock-outs in a difficult-to-study mammal, but also in using the methodology to knock-out three of the genes shown to be important in mice preimplantation development. The study highlights important differences in the drivers of preimplantation development between mice and cattle which makes this work very interesting and important.

**Response: Thank you very much for taking time to review this manuscript.**

There are though some minor but rather serious mistakes and a small part of the data is unconvincing.

In the abstract the authors say SOX2 is dispensable for OCT4 and NANOG but mean indispensable!!

**Response: Thank you for pointing out the mistake. We have corrected it in the revised version.**

In the section “Base editors enable…”, it would be worthwhile to also indicate the % editing for each of the three genes for both methods.

**Response: Thank you for the suggestion. We have added the information as “****Moreover, the percentages of editing for *SMAD4*, *TEAD4* and *CDX2* by BE3 are 54.8%, 32.3% and 54.8%, respectively and those by ABE7.10 are 76.7%, 33.3% and 93.3%, respectively.”.**

The section on OCT4 knock-out should have the supplementary figure moved into the main paper. Why were the shown FigS3I and J panels not discussed in the text? It appears that Sox2 protein is upregulated/maintained upon OCT4 knock-out. This is quite important data.

**Response: Thank you for the advice and comment. We have moved the figure into the main paper as Fig 1 and added the related description into the text.**

**As for the issue on SOX2 protein, we have checked our data analysis** **and found there is no significant difference in the signal intensity of SOX2** **for those SOX2 positive cells between control and KO groups. However, SOX2 is sustained in the trophectoderm cells, which could be attributed to the developmental delay of *OCT4* KO blastocysts.**

The section on Sox2 expression in bovine early embryos should probably include a reference to ref.6 as this is not all that novel.

**Response: Thank you for the suggestion. We have included the reference in the updated manuscript.**

The incorrect figure is quoted in line 155. Also, it was not shown in Fig 5A that the number of ICM cells was decreased! This data needs to be added.

**Response: Thank you for the comment and suggestion. We have added the data into Fig 5A.**

The immunofluorescence of Figure 5 is in some instances unconvincing, even when viewed at magnification on a screen. In particular, the highly significant down regulation of OCT4 protein at E7.5 is difficult to believe. Maybe the authors can linearly adjust the histograms on the confocal (equally for KO and Control) to allow better visualization.

**Response: Thank you for the suggestion. We have adjusted the brightness and contrast in the current version. The significant downregulation of OCT4 at E7.5 is highly reproducible as shown by other examples in Fig R1.**

Indeed the CDX2 intensity reduction in Fig 5A looks more real but is claimed to be non-significant.

**Response: We have re-examined the data analysis and found there is no significant difference in the CDX2 intensity.**

In Fig 5C NANOG staining is so weak even in controls as to be deemed background, making the reduction unconvincing.

**Response: Thank you for this comment. We have adjusted the brightness (equally for KO and Control) of the picture to allow better visualization. Moreover, here are more pictures showing NANOG reduction in *SOX2* KO blastocysts (Fig R2).**

The statement in lines 185-7 is too sweeping. These results do not substantiate that SOX2 is necessarily upstream of OCT4 and NANOG. If loss of SOX2 wipes out the ICM cells as claimed this will also remove NANOG and OCT4 expression in these cells, whether SOX2 protein controls their expression or not.

**Response: Thank you for the insightful comment. We have revised the sentence as “These results collectively suggest that SOX2 is required for maintaining correct expression profile of NANOG and OCT4 in bovine blastocysts.”.**

The CDX2 mosaic experiment is lovely, well done, and also well interpreted in line 254, that the suppression is developmental context-dependent.

In the methods section, please provide details of microinjector apparatus and procedure.

**Response: Thank you for the comment and suggestion. We have added the information into the methods section as “10-20 pL mixture of 100 ng/µL sgRNA and 200 ng/µL ABE7.10 or BE3 mRNA were microinjected into bovine zygotes at 12 h post insemination (hpi) by using a micromanipulator (TransferMan, Eppendorf, Germany). Control embryos were injected with the same amount of mRNA without sgRNA. To maximize the editing efficiency of the gene of interest, a cocktail of two or three sgRNAs were microinjected together with ABE7.10 or BE3 mRNA. Each sgRNA was kept at the same concentration (100 ng/µL). For constructing CDX2 mosaic embryos, we microinjected base editor components into one blastomere at 2-cell stage.”.**

When co-injection several sgRNA’s indicate whether each was kept at the same concentration.

**Response: Yes, each sgRNA was kept at the same concentration (100 ng/µL) when co-injection was performed.**

For genotyping, was this done after immunofluorescence and how were mosaic embryos identified (Fig 3E etc)? Indicate nested PCR conditions.

**Response: As for genotyping, every embryo was recovered after the immunofluorescence and genotyping performed as published previously (Frum *et al.* 2019, Daigneault *et al.* 2018). Then we identified the mosaic embryos according to both immunofluorescence and genotyping analysis.**

**As for nested PCR, two rounds of PCR were performed by using primeSTAR HS DNA Polymerase (Takara, Cat. #R040A). PCR condition was: 98°C for 2 min followed by 35 cycles of 98°C for 10 s, 60°C for 5 s, 72°C for 1.5 min, and a final 5-min step at 72°C .**

I would also put something in the abstract to indicate that you have knocked-out three genes with your methodology.

**Response: Thank you for the suggestion and we have included this information into the revised abstract.**

Line 46 “by the” instead of “of the”

**Response: We have corrected the mistake.**

Line 54, references missing

**Response: We have inserted the references.**

Line 126: greatly

**Response: We have corrected the mistake.**

Line 146: codon

**Response: We have corrected the mistake.**

Line 155: word order, use “decreased rather dramatically”

**Response: We have corrected the mistake.**

Reviewer 2: This manuscript by Luo and colleagues examines the regulation of gene expression in early bovine embryos. Generally speaking, the study of non-rodent mammalian development is significant because developmental mechanisms can differ between rodents and humans. By contrast, some developmental mechanisms may be more similar between cow and human during preimplantation, thus meriting the study of bovine preimplantation gene regulatory mechanisms.

**Response: Thank you very much for taking time to review the manuscript.**

The authors use CRISPR-mediated base-editing to investigate the roles and regulation of SOX2 in the cow embryo. Published studies have shown that SOX2 is an early pluripotency marker in mouse, but not in human or cow. Overall, the novelty of the study is not as high as desired because other groups have characterized the expression pattern of SOX2 in cow preimplantation embryos (Gerri et al., Nature 2020) and have evaluated the consequences of Sox2 knockout in this same context (Simmet et al., PNAS).

**Response: Although it is not the major novelty of the paper, the present study specifically addressed the expression pattern and dynamics of SOX2 in bovine early, medium and late blastocyst while Gerri et al.’s study have only presented data on expanded blastocysts (Gerri *et al.* 2020).**

**To our knowledge, there has been no study evaluating the consequences of *SOX2* knockout in bovine blastocysts. In fact, the study by Simmet et al. has investigated the consequence of *OCT4* knockout rather than *SOX2* knockout in bovine blastocysts (Simmet *et al.* 2018).**

Some novelty is provided by 1) use of base editing (as opposed to reliance on CRISPR-induced indels) in cow embryos, 2) RNA-seq analysis of Sox2 knockout embryos, and 3) evaluation of CDX2 as an upstream regulator of the Sox2 expression pattern. However, not all of these directions are supported by appropriately rigorous study. As detailed below, the RNA-seq analysis pipeline is superficial and error-prone; the Cdx2 phenotype is weak (very modest affects on Sox2 expression).

**Response: Thank you for the comment. We have answered these questions as below.**

Admittedly, additional novelty is provided by the observation that Sox17 expression is lowered in the absence of Sox2, indicative of either a) a novel cell-autonomous role for Sox2 in promoting expression of Sox17 or b) a conserved non cell-autonomous role for Sox2 in promoting expression of Sox17. However, these mechanistic possibilities are not explored. All in all, the manuscript falls short of making a big splash.

**Response: Thank you for the comment. The main objective of the present study was to examine the functional consequence of *SOX2* knockout in bovine blastocysts. Because we found *SOX2* knockout reduced NANOG in bovine blastocysts, indicating a failure of the second lineage specification event to form epiblast and primitive endoderm, we thus verified if this was true by using an primitive endoderm marker, SOX17. Indeed, we found SOX17 protein level was also reduced. While we admitted that the result is interesting, it is beyond the scope of the present study but warrants further investigation in the future.**

Figure 1 could be moved to supplemental or cut entirely. This level of analysis is only really relevant to the study of the gene knockouts studied in this paper (Sox2 and Cdx2) – rather than Smad4, Tead4, and Cdx2).

**Response: Thank you for the suggestion and we have moved it into Fig S1.**

Figure 2 is redundant with data already described by Gerri et al., Nature 2020

**Response: We prefer to keep the data as the present study specifically addressed the expression pattern and dynamics of SOX2 in bovine early, medium and late blastocyst while Gerri et al.’s study have only presented data on expanded blastocysts (Gerri *et al.* 2020).**

Figure 3 includes multiple pairwise comparisons (t-tests for 3 samples at a time) – leading one to question the reported significance.

**Response: Sorry for the mistake. We used one-Way ANOVA to analyze significant differences among three groups and two-tailed unpaired student t-tests to compare differences between two groups. We have updated the methods section accordingly.**

Figure 4 also relies on t-tests for RNA-seq data, when false discovery rate is the standard. Additionally, the RNA-seq data do not take into account whether the Sox2 ko embryos could be developmentally delayed and whether this could explain the reported gene expression differences.

**Response: As for RNA-seq analysis, we did use false discovery rate (FDR) rather than t-tests. For differential expression analysis of RNA-seq data, the differential expressed genes were identified using following threshold: Padj<=0.05 and Foldchange (KO/WT)>=2 for up-regulated genes; Padj<=0.05 and Foldchange (KO/WT)<=0.5 for down-regulated genes. Here Padj referred to FDR adjusted P value, which has been corrected by BH method based on Wald test P value. To avoid any confusion, we have presented this information in detail in the methods section.**

**To address the concern on development delay of SOX2 KO blastocysts, we collected RNA-seq samples at early blastocyst stage prior to the emergence of phenotypical difference. Indeed, as shown in Fig S4, the blastocysts from both control and KO groups are similar in size. Moreover, we have performed the principal component analysis and clustering analysis by comparing the transcriptomes of wildtype (WT BL) and SOX2 KO (KO BL) blastocysts with the one of wildtype late morula (WT MO), which was previously generated in our lab. Results show that SOX2 KO BL cluster together with WT BL rather than WT MO (Fig R3). Collectively, these data suggest the observed gene expression changes are not due to developmental delay.**

Figure 5 – why is the CDX2 level reduced in the SOX2 KO in panel A? Why isn’t OCT4 detectable in WT panel B or NANOG detectable in WT panel C? How was the relative intensity quantified?

**Response: We have checked our data analysis and found there is no significant difference on CDX2 level. It is probably due to the variability between samples.**

**We have adjusted the brightness (equally for KO and Control) of the picture to allow better visualization in panel B and C.**

**Regarding the relative intensity, the nuclear region was encircled based on the DAPI signal and the intensity measured. The same region was moved to the cytoplasm area and background intensity obtained. The specific signal was calculated by subtracting the cytoplasmic intensity from the nuclear intensity. Finally, the data were normalized to the relative channels in control groups.**

Figure 6 – Panel A is attempting to show that CDX2 is responsible for repressing expression of SOX2 in the trophectoderm, but SOX2 is barely visible in a few CDX2 KO trophectoderm cells (likewise for panel E). In panels B, C, the SOX2 expression level in WT and CDX2 KO is quantified in terms of intensity – was this measured for all trophectoderm cells? A subset?

**Response: Yes, the SOX2 intensity was measured for all trophectoderm cells. Meanwhile, the experiment described in panel A was performed seven times and the results were highly reproducible. To further test if CDX2 is responsible for repressing SOX2, we performed the analysis of CDX2 mosaic embryos (panel E) and similar conclusion was obtained.**

Reviewer 3: This is an important paper on comparative developmental biology of mammalian embryos. The results are important and supported by the experimental data. The paper rises to the level of importance expected for PLOS Genetics because of its importance to understanding how fundamental mechanisms of development have diverged between species.

**Response: Thank you very much for evaluating our manuscript.**

All of my comments are very minor. The paper is well written but could use some editing for English. Moreover, the title and abstract does not do justice to the paper since little is said about the impact of the OCT4 knockout and the abstract does not make clear why CDX2 is believed to regulate SOX2 expression.

**Response: Thank you for the advice and suggestion. We have edited the English writing in the updated manuscript.**

**As suggested, we have added the information on *OCT4* knockout and the regulation of CDX2 on SOX2 into the abstract.**

Here are the minor comments:

Line 40 (remarkably) spelling

**Response: We have corrected the mistake.**

Line 46 – reword this sentence. Perhaps “begins with the first sequential cell fate decisions occurring in close temporal relationship to each other”.

**Response: Thank you for the suggestion. We have reworded the sentence.**

Line 80 – change to “highly-efficient”

**Response: We have corrected the mistake.**

Line 115 – change to “tested”

**Response: We have corrected the mistake.**

Line 133 – change to “be expressed”

**Response: We have corrected the mistake.**

Line 196 – change to “out of”

**Response: We have corrected the mistake.**

Methods – don’t capitalize names of chemicals (sodium pyruvate etc.)

**Response: We have corrected them.**

Line 310 – no space between degree symbol and number

**Response: We have corrected the mistakes.**

Tables 1 and 2 – italicize gene symbols

**Response: We have corrected the mistakes. Thank you very much again.**

**References**

1.	Frum T, Watts JL, & Ralston A (2019) TEAD4, YAP1 and WWTR1 prevent the premature onset of pluripotency prior to the 16-cell stage. *Development* 146(17).

2.	Daigneault BW, Vilarino M, Rajput SK, Frum T, Smith GW, & Ross PJ (2018) CRISPR editing validation, immunostaining and DNA sequencing of individual fixed bovine embryos. *Biotechniques* 65(5):281-283.

3.	Gerri C, McCarthy A, Alanis-Lobato G, Demtschenko A, Bruneau A, Loubersac S, Fogarty NME, Hampshire D, Elder K, Snell P, Christie L, David L, Van de Velde H, Fouladi-Nashta AA, & Niakan KK (2020) Initiation of a conserved trophectoderm program in human, cow and mouse embryos. *Nature* 587(7834):443-447.

4.	Simmet K, Zakhartchenko V, Philippou-Massier J, Blum H, Klymiuk N, & Wolf E (2018) OCT4/POU5F1 is required for NANOG expression in bovine blastocysts. *Proc Natl Acad Sci USA* 115(11):2770-2775.
