# Supplementary material for: Base editing in bovine embryos reveals a species-specific role of SOX2 in regulation of pluripotency
Source: PLoS Genet. 2022 Jul 5;18(7):e1010307. doi: 10.1371/journal.pgen.1010307 (PMC9286228; doi:10.1371/journal.pgen.1010307)
Supplement: Attachment — Submitted filename: Response to Reviewers final.docx [file pgen.1010307.s014.docx]

**Dear Dr. Marisa S Bartolomei and Dr. Gregory Barsh,**

**Thank all reviewers and editors for their valuable comments and suggestions. The specific changes made in the revised manuscript are summarized in our point-by-point responses as shown below.**

Editors’ Comments to the Author:

The manuscript was fully evaluated at the editorial level and by the same reviewers as the first round. While two of the reviewers were satisfied with the revision, one reviewer is still concerned with staging and the potential for the mutations to result in embryonic delay, which compromises the interpretation of the phenotype. Based on the reviews, we will not be able to accept this version of the manuscript, but we would be willing to review a revised paper if you can address all of the concerns of the reviewer. **Response: Thank you very much. We have addressed the concern on staging and the potential for the mutations to result in embryonic delay specifically as below.**

Reviewers’ Comments to the Author:

Reviewer #1: I thank the authors for answering all my queries well and presenting additional evidence for the IF results.

**Response: Thank you very much for taking time to review this manuscript.**

Reviewer #2: I have reviewed the authors’ rebuttal, but I still have concerns about the revised manuscript, which I delineate below. I consider these to be major concerns, because they pertain to the paper’s main big points. Therefore, I am not confident in the quality of this study in its current form.

• As I mentioned in the first round of review, the SOX2 time course was already shown in Gerri et al., 2020, Extended Data Fig. 5C: https://www.ncbi.nlm.nih.gov/pmc/articles/PMC7116563/figure/F8/

The authors argue that they show more time points than Gerri et al., which is true. However, those additional time points encompass stages when SOX2 is not expressed (prior to 8-cell stage). Therefore, this aspect of the manuscript is a more incremental contribution to our understanding of bovine preimplantation.

**Response: Thanks for your recognition.**

• I previously raised concerns about embryo staging. Throughout the paper, the authors are inconsistent in their definitions of stage, sometimes reporting cell counts, but usually relying on surrogates for determining stage, such as time in culture (e.g., E7.5, E8.5), embryo size, or subjective terms (e.g., morula, early blastocyst, late blastocyst). This issue is not resolved, and complicates interpretation of phenotypes, detailed below.

**Response: Thanks for your comment. Based on the suggestion and previous studies on bovine embryos (Simmet *et al.* 2018), we have unified the embryo staging by using time in culture (e.g. day 7.5). By the way, it should be reminded that the developmental asynchrony emerges upon blastocyst formation (around D7.5 in our system) for typical bovine embryo in vitro culture system. As seen in Fig. R1, blastocysts with various size are observed in both D7.5 and D8.5. However, early (arrowheads) and middle blastocysts (arrows) are predominant on D7.5 while late blastocysts (asterisks) are predominant on D8.5. Thus, we have to consider both time in culture and embryo size for blastocyst stage. We have presented the specific information for specific experiments in the revised manuscripts.**

• CDX2 KO phenotype: does CDX2 repress SOX2 in the TE? In the first round of review, I raised the concern that the CDX2 phenotype could be a result of developmental delay. Since the CDX2 phenotype is the perdurance of SOX2 in the TE slightly longer than what is observed normally. The authors show nicely in a wild type time course in Fig. 2C,D that SOX2 is normally gradually diminished in the TE. They should show their data in this way for the CDX2 KO embryos.

Therefore, I cannot tell if the CDX2 KO and WT embryos are stage-matched and whether the reported phenotype (SOX2 perdurance) is “real.”

**Response: Yes, our results suggest CDX2 does repress SOX2 in the TE. As shown in Fig. R2 based on your suggestion, we have presented our data in the revised manuscript. Results show SOX2 intensity is normally gradually diminished in the TE in WT blastocysts whereas it is perdured in CDX2 KO blastocysts. Importantly, our mosaic experiment (Fig. 6D and 6E) clearly demonstrated CDX2 KO TE cells exhibit higher SOX2 compared with WT TE cells in the same blastocysts (stage-matched control), ruling out the possibility of developmental delay. Therefore, we proposed CDX2 indeed inhibits the expression of SOX2 in TE cells.**

• Same topic, different KO: the authors show in Fig. 3G,H that total cell numbers are reduced in SOX2 KO embryos (by half, which is a pretty dramatic difference). Is this a developmental delay or are cells dying?

**Response: Thanks for the comments. We speculate SOX2 is required for cell proliferation after blastocyst formation while no difference was found on blastocyst rate between WT and KO groups. Moreover, as shown in Fig. 5B, OCT4 is still present in all blastomeres in KO blastocysts (although at a reduced level relative to WT), suggesting cells are not dying. However, the related mechanism has yet to be explored.**

Do embryos have the same number of inside and outside cells? The authors look at the number of CDX2-positive and -negative cells (Fig. 5A), but this is gene expression, and not lineage allocation per se.

**Response: Thanks for the comments. To determine the inside and outside cells number, we used CDX2 (a TE marker) to assess effects of SOX KO on ICM/TE lineage specification. This is because OCT4 is universally expressed in bovine blastocysts (in contrast with mouse) and NANOG is also affected by SOX2 KO, we do not have a reliable maker of ICM to determine ICM lineage directly.**

This raises questions as to whether E7.5 WT and KO embryos are appropriate comparisons for the RNAseq or immunofluorescence experiments shown in Fig. 4-5 and conclusions drawn about regulation of OCT4 and NANOG expression.

Rather, the authors argue that the embryos are the same stage because they are the same size (Fig. S5C). But size is not a reliable indicator of developmental stage during preimplantation, when cleavage divisions and the dynamic and iterative process of blastocoel expansion complicate interpretation of embryo size. I appreciate Fig. R3, as an indicator that they can resolve transcriptional differences between morula and blastocyst, but I can’t tell if this is sufficiently granular to address my concerns. In addition, these data do not serve as a good control if they are not included in the revised manuscript.

**Response: Thanks for the insightful comments. Because our developmental data already show *SOX2* KO does not affect blastocyst development, we then collected early blastocysts with similar size on D7.5 to perform RNA-seq. To further address the concern on developmental stage, we collected similar blastocysts with those samples used for RNA-seq and counted total cell numbers. As shown in Fig R3A, *SOX2* KO blastocysts show no evident difference in terms of total cell number compared with WT embryos. Thus, it is appropriate to collected D7.5 early blastocysts for RNA-seq.**

**Moreover, as shown in first round of review, we have performed the principal component analysis and clustering analysis by comparing the transcriptomes of wildtype (WT BL) and *SOX2* KO (KO BL) blastocysts with the one of wildtype D6 morula (WT MO), which was previously generated in our lab (Datasets: GSE158679). Results show that *SOX2* KO D7.5 BL cluster together with WT D7.5 BL rather than WT D6 MO (Fig R3B and R3C). We have included this result in the revised manuscript (Fig. S4E and S4F).**

**Last but not least, to directly determine if *SOX2* KO affect NANOG and OCT4 at earlier stage prior to blastocyst formation, we performed IF analysis on D6.5 and found OCT4 and NANOG were both reduced in *SOX2* KO embryos (Fig. R4, 5C-5E). Therefore, this result consolidates our conclusion that SOX2 is required for proper expression of NANOG and OCT4 in bovine embryos.**

Reviewer #3: This is a very good and important paper.

**Response: Thank you very much for taking time to review this manuscript.**

**Reference**

1.	Simmet K, Zakhartchenko V, Philippou-Massier J, Blum H, Klymiuk N, & Wolf E (2018) OCT4/POU5F1 is required for NANOG expression in bovine blastocysts. *Proc Natl Acad Sci USA* 115(11):2770-2775.
